# The Benefits of Resistance Training in Obese Adolescents: A Systematic Review and Meta-analysis

**DOI:** 10.1186/s40798-022-00501-3

**Published:** 2022-09-04

**Authors:** Bruno Ribeiro, Pedro Forte, Raquel Vinhas, Daniel A. Marinho, Luís B. Faíl, Ana Pereira, Fernando Vieira, Henrique P. Neiva

**Affiliations:** 1 Department of Sport Sciences, University of Beira Interior, Convento de Santo António, 6201-001 Covilhã, Portugal; 2 Research Center in Sports Sciences, Health Sciences and Human Development, CIDESD, 6200-151 Covilhã, Portugal; 3 Instituto Politécnico de Bragança, Bragança, Portugal; 4 Higher Institute of Educational Sciences of the Douro, Penafiel, Portugal; 5 NOVA School of Science and Technology, Universidade NOVA de Lisbon, Lisbon, Portugal; 6 Department of Science and Technology, Polytechnic Institute of Setubal, 2910-761 Setúbal, Portugal; 7 KinesioLab – Research Unit in Human Movement, Piaget Institute, Lisbon, Portugal; 8 RECI – Research Unit in Education and Community Intervention, Piaget Institute, Lisbon, Portugal; 9 ISEIT de Almada, Piaget Instituto, Lisbon, Portugal

**Keywords:** Strength-training, Body mass, Fat, Fitness, Youth

## Abstract

**Background:**

Physical activity is essential in acquiring healthy lifestyle behaviors in the early years of maturational development and preventing various diseases. Resistance training (RT) is fundamental for improving body composition and is increasingly recommended for obese adolescents. A systematic review and meta-analysis were performed to synthesize and analyze data on the effects of RT programs in this population, seeking to develop useful recommendations for health and sports professionals.

**Methods:**

A search was performed using four databases (Web of Science, Scopus, PubMed, and ScienceDirect). According to specific inclusion criteria, twenty-one studies were selected to evaluate the impact of RT on body mass index (BMI), body fat, waist circumference, muscle strength, insulin sensitivity, lean mass and cardiorespiratory fitness.

**Results:**

After the RT programs, the adolescents improved their muscle strength (SMD, 1.44; 95% CI: 0.76–2.12), cardiorespiratory fitness (SMD, 1.09; 95% CI: 0.15–2.04), BMI (SMD, 0.21; 95% CI: 0.07–0.35), waist circumference (SMD, 0.27; 95% CI: 0.06–0.47) and body fat (SMD, 0.20; 95% CI: 0.05–0.34). However, insulin sensitivity (SMD, 0.32; 95% CI: − 0.47 to 1.10) and lean mass (SMD, 0.12; 95% CI: − 0.06 to 0.31) did not reveal any changes. Different RT programs were used but it seems that 2–3 times/week ∼60 min/session of RT for 12 weeks should be recommended for positive changes.

**Conclusions:**

RT seems to be effective when the objective is to improve muscle strength, cardiorespiratory fitness and can be an efficient strategy to reduce obesity in adolescents by reducing body fat, waist circumference and body mass index.

PROSPERO Registration number: CRD42022333411.

## Key Points

1. There is a tendency for participants in the RT programs to be enthusiastic and enjoy the exercise intervention, which can be an alternative exercise modality for obese adolescents.
2. Interventions that used protocols 2–3 times/week ∼60 min/session during 12 weeks seem to show more positive effects in obese adolescents (i.e., decreased body fat, waist circumference and body mass index; increased muscle strength and cardiorespiratory fitness).
3. RT programs aimed at reducing body fat in obese adolescents should not neglect the need to be combined with a nutritional plan to be effective.

## Background

In adolescence, primordial biological changes occur and we acquire habits that will influence the entire course of our lives [1]. During this period, behaviors related to physical activity and healthy eating habits directly affect body composition and health [2]. These factors, when improperly oriented, can promote a sedentary lifestyle, increase body weight, and induce health problems such as obesity [3]. Although genetic factors influence the development of obesity, environmental factors are directly associated with its exponential increase in adolescents [4]. These are related to metabolism, dyslipidemias and circulatory diseases or insulin resistance [5–8]. Furthermore, others have suggested that obese adolescents are more likely to uncover symptoms of depression, anxiety, and compulsive eating behaviors, thus being advised to consult psychopathology specialists [9].

Physical activity is essential for young people diagnosed with obesity, and aerobic training (AT) is usually suggested to reduce obesity levels and improve overall health [10–13]. However, AT may not be well tolerated by obese people, and excess weight limits the intensity at which exercise can be performed, often causing withdrawal from training [14, 15]. Hence, resistance training (RT) can be an alternative to AT. Furthermore, the increased body mass of obese adolescents might be an advantage for performing resistance exercises [16, 17]. There was an increase in interest regarding RT performed by obese young people [18]. Some findings suggest that RT may be essential to improve sports performance and physical conditioning, and also to control body composition [8, 16, 19]. RT contributes to the maintenance or increase of muscle mass, increasing the resting metabolic rate and potentially increasing daily caloric expenditure [20].

Researchers have tried to better understand the effect of RT in adolescents in recent years. Schranz et al. [21] found that RT improved self-esteem, and Kim [22] analyzed the effect of RT in 13 adolescents and found a significant decrease in waist circumference and an increase in cardiorespiratory fitness. Also, some authors reported that, when guided by qualified professionals and applied at stages of maturational development, RT improves muscle strength in programs between 8 weeks [23] and 22 weeks [24]. Other evidence from RT protocols indicates better injury prevention during sports and enhancement of motor performance [25, 26] and a positive health effect through improving lipid profile [27]. Additionally, RT appears to have a direct relationship with increased bone mineral density [28] and improved resting blood pressure [8] without affecting growth [29]. Also, Shaibi et al. [30] showed significant improvements in insulin sensitivity levels, after 16 weeks of RT. Despite the positive results obtained after RT, it is still neglected in weight loss programs, over AT programs [31, 32]. This may be because some findings have been controversial about the effects on body composition, showing detrimental results in reducing body fat [21, 33, 34].

As far as we know, there is a lack of literature attempting to summarize the main findings on the effects of RT in obese adolescents and further studies are recommended for clear conclusions [35]. Therefore, it is necessary to clarify the magnitude of the contribution of physical activity, namely isolated RT, in the treatment of obesity and associated diseases. Thus, it is essential to synthesize and analyze the main results of using RT to improve the health of obese adolescents, analyzing specific variables such as BMI, body fat, lean mass, waist circumference, muscle strength, cardiorespiratory fitness, and insulin sensitivity.

## Methods

This review aimed to summarize the findings and conclusions reported in the literature on the effect of RT in adolescents. An extensive literature search was developed to identify published articles on the subject. The articles selected for the meta-analysis met the inclusion criteria.

### Search Strategy

PRISMA guidelines guided the systematic review (Preferred Reporting Items for Systematic Reviews and Meta-Analyses) [36]. The search was performed using the Boolean method, including the AND/OR operators only for studies that contained relevant key terms. Original research articles that identified the effects of resistance training on body composition were analyzed. The research was performed up to May 6, 2022, in four databases (Web of Science, Scopus, PubMed, and ScienceDirect) using the keywords: ("adolescence OR teenager") AND ("resistance training OR resistance exercise") AND ("obesity OR loss of weight") in vast combinations and without year or language limitations. Review articles (qualitative review, systematic review, and meta-analysis) were not included. Furthermore, we only included studies that used resistance training alone, with no other forms of exercise. After the exclusion of duplicates, the title and abstracts were read, with non-relevant articles excluded. The articles were selected after complete reading according to the inclusion and exclusion criteria. This process was carried out by two reviewers to reduce the risk of bias.

### Inclusion and Exclusion Procedures

The inclusion criteria for this study were as follows: (1) experimental interventions including an isolated RT program; (2) results of the effects of RT on BMI, body fat, lean mass, cardiorespiratory fitness, insulin sensitivity, waist circumference, and muscle strength; (3) obese or overweight adolescents aged 18 years and under; (4) studies had to include data referring to the variables analyzed before and after the intervention program; (5) include at least one intervention group and a control group. The exclusion criteria were as follows: (1) literature reviews; (2) other training programs than RT isolated; (3) studies with different variables to those listed in inclusion criterion (2) above; (4) aged over 18 years old; (5) studies that did not analyze the variables before and after the intervention program.

### Quality Assessment

To proceed with the analysis of the methodological quality of this review, two independent reviewers prepared their analysis following the methods and domains proposed by The Cochrane Collaboration [37], allowing the identification of the risk of bias in each study according to (1) Generation of Random Sequence; (2) Concealment of allocation; (3) Blinding of participants and professionals; (4) Blinding of outcome evaluators; (5) Incomplete outcomes; (6) Report of selective outcome; (7) Other sources of bias. For these criteria, the following classifications were used: high risk of bias, low risk of bias, and uncertain risk of bias. Review Manager software (RevMan, The Nordic Cochrane Center, Copenhagen, Denmark Version 5.4) was used to create the risk of bias graphs. In case of disagreement about the quality of the studies, a third reviewer was consulted.

### Data Extraction and Analysis

Relevant data were extracted from the included studies, such as participants, age, intervention period, number of sessions and duration, details of the interventions, and their effects compared to control groups. The variables analyzed in the articles (i.e., muscle strength, body mass index, cardiorespiratory fitness, waist circumference, lean mass, body fat, insulin sensitivity) were extracted and are detailed in the results. The meta-analysis to determine the effect of the RT programs in adolescents with obesity was conducted using Review Manager software (version 5.4; The Nordic Cochrane Centre, The Cochrane Collaboration, Copenhagen, Denmark). The effects of the RT program on adolescents were analyzed regarding muscle strength, body mass index, body fat, lean mass, waist circumference, insulin sensitivity, and cardiorespiratory fitness. There was a control group in all of the studies. The means and standard deviation from pre- and post-intervention were used to determine the intervention effect, which was calculated using the standardized mean difference (SMD) for each study and 95% confidence intervals (95% CI). Results were considered statistically significant at *p* < 0.05, and to classify the magnitude of the intervention effect the category of Cohen was selected (*d* values between 0.2 and 0.5 represent a small effect size; between 0.5–0.8 a medium effect size; greater than 0.8 a large effect size) [38]. Regarding the effects of RT, intervention benefits from negative changes in BMI, body fat, and waist circumference and positive changes in muscle strength, lean mass, cardiorespiratory fitness, and insulin sensitivity.

## Results

**Fig. 1:**
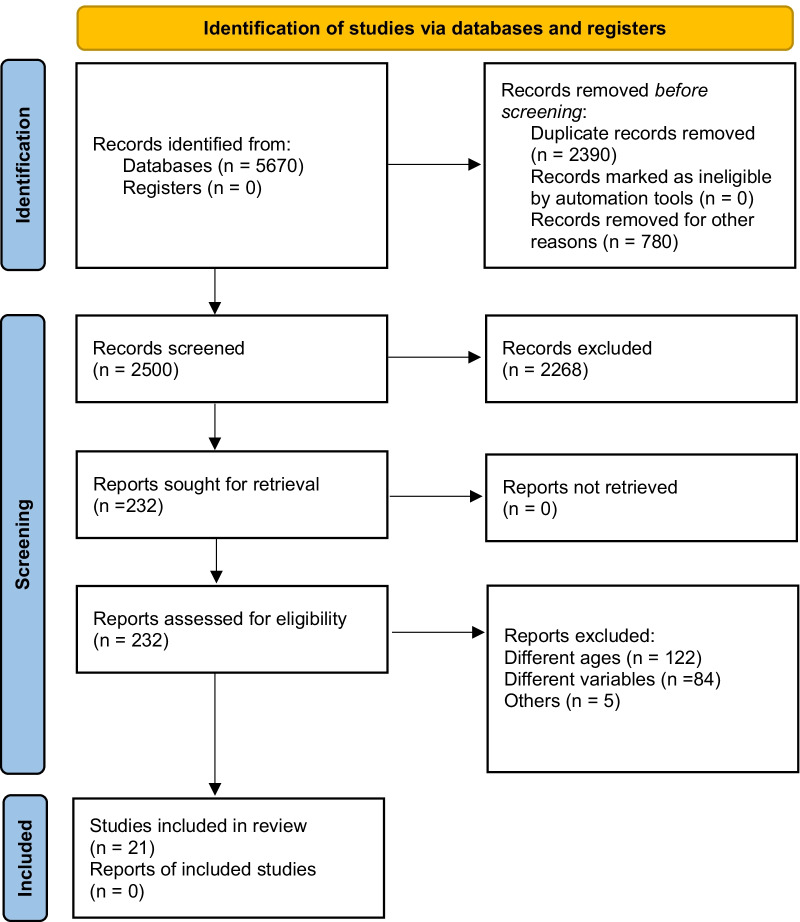
PRISMA (Preferred Reporting Items for Systematic Reviews and Meta-Analyses) flowchart for study identification

The initial search included 5670 articles, of which 2500 potentially relevant articles remained after excluding duplicate studies. Then, 2268 studies were excluded after screening the titles and abstracts. The remaining 232 full-text articles were assessed for eligibility, of which 211 articles were excluded for various reasons, meaning 21 articles were included in the analysis of the effects of RT in obese or overweight adolescents. A flowchart outlining the identification and selection of studies focusing in the effects of RT in obese adolescents is provided in Fig. 1.

**Fig. 2:**
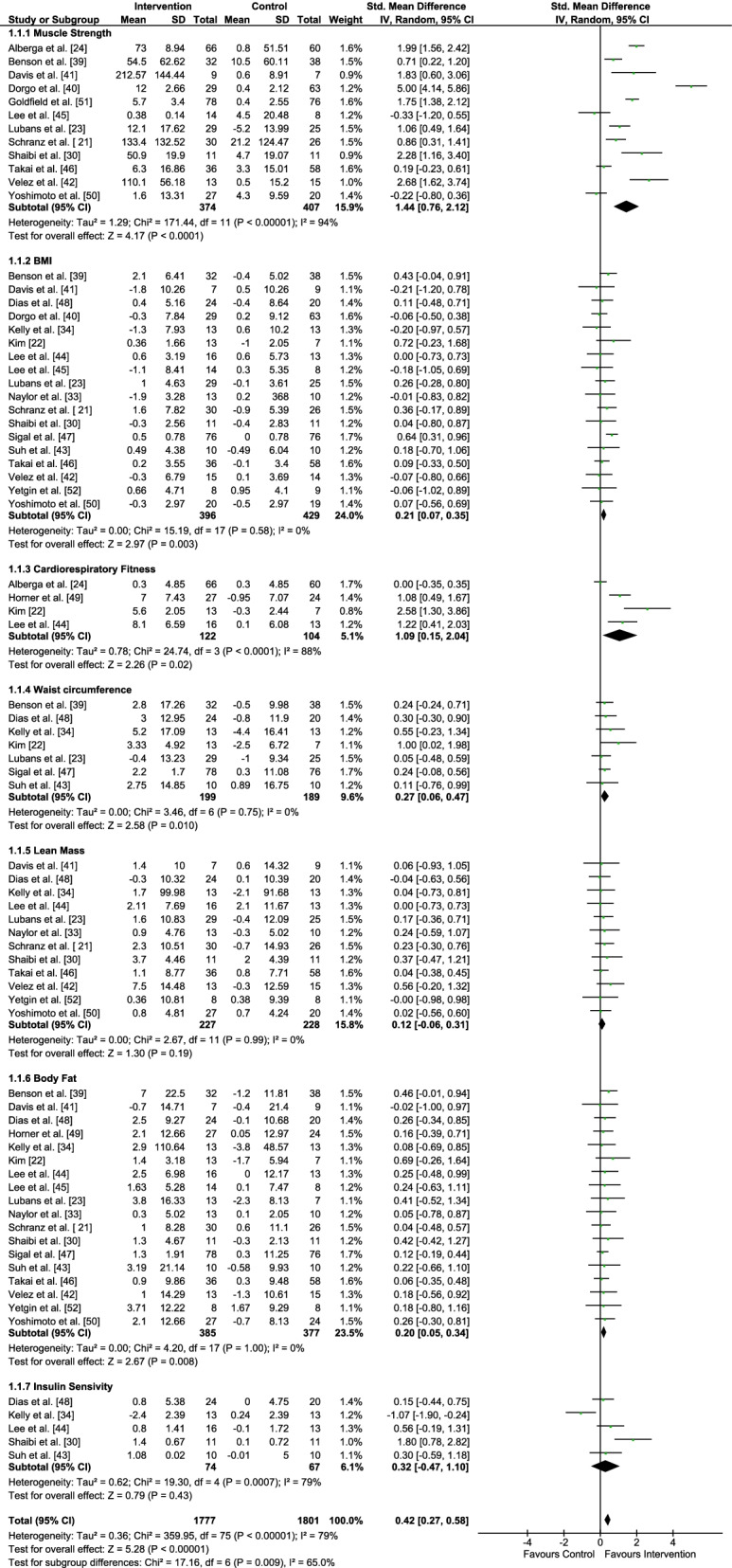
Forest plot of comparison for muscle strength, body mass index (BMI), cardiorespiratory fitness, waist circumference, lean mass, body fat and insulin sensitivity. The center of each square represents the standard mean difference for individual trials, and the corresponding horizontal line stands for 95% confidence interval (CI). The diamonds represent pooled results

Table 1 summarizes the studies that investigated the effects of the RT program and Fig. 2 provides the meta-analysis of the studies included. Body fat and BMI were analyzed in eighteen studies [21–23, 30, 33, 34, 39–49, 52]. However, Horner et al. [49] only evaluated body fat and Dorgo et al. [40] evaluated BMI. Body fat was reduced in most studies [23, 30, 39, 43–45, 48, 50] with an article reporting medium effect [22]. Some studies were not able to demonstrate any change in this variable after a RT program [21, 33, 34, 42, 46, 47, 49, 52]. Data referring to BMI indicated that RT induces a small reduction, according to three studies [21, 23, 39]. However, in fourteen studies, there were no changes in this variable [30, 33, 34, 40–48, 50, 52]. Nevertheless, Kim [22] and Sigal et al. [47] reported a medium effect. Three of the twelve studies analyzing lean mass verified a small increase [21, 30, 33], with results showing that RT increased significantly in one of them [42]. However, no variation was found in eight studies [23, 34, 41, 44, 46, 48, 50, 52]. Insulin sensitivity was assessed in five studies and one of them found a large [30] and two a medium variation [34, 44]. RT did not change waist circumference in three studies [23, 43]. However, small positive changes [39, 47, 48] and large effects [22] were shown. The effects of RT on cardiorespiratory fitness were assessed in four studies [22, 24, 44, 49], with large improvements shown, except for one study [24]. Specifically, the effect of RT on muscle strength induced a medium increase in one [39] and a large increase in eight studies [21, 23, 24, 30, 39–42, 51]. Only the studies by Takai et al. [46], Lee et al. [44] and Yoshimoto et al. [50] did not reveal any changes.

**Table 1:** Description of the studies of the effects of RT included in the analysis

Study	Sample Program duration	Age (y)	RT	Outcomes
Schranz et al. [21]	30 (RT) 26 (C) 144 sessions (48 wks)	14.9 ± 1.4 15.1 ± 1.6	10 exercises 3 × 10RM	↑MS ↓BMI ↔BF ↑LM
Kim et al. [22]	13 (RT) 07 (C) 32 sessions (16 wks)	14.7 ± 0.4 15.0 ± 0.6	10 exercises 50–60%1RM 1–2 sets × 12	↓BF ↓BMI ↓WC ↑CRF
Lubans et al. [23]	15 (RT) 16 (C) 16 sessions (8 wks)	14.9 ± 0.6 14.5 ± 0.6	10 exercises Wks 1–4 2 × 8–12 Wks 5–8 8–10	↓BF ↓BMI ↔WC ↑LM ↑MS
Alberga et al. [24]	78 (RT) 76 (C) 88 sessions (22 wks)	15.9 ± 1.5 15.6 ± 1.3	07 exercises 3 × 6–15RM	↑MS ↔CRF
Shaibi et al. [30]	11 (RT) 11 (C) 32 sessions (16 wks)	15.1 ± 0.5 15.6 ± 0.5	10 exercises Wks 1–4 62–71% 1RM 1 × 10–15 Wks 5–10 74–88%1RM 2 × 3–15 Wks 11–16 92–97%1RM 3 × 8–12	↑LM ↓BF ↔BMI ↑MS ↑IS
Naylor et al. [33]	13 (RT) 10 (C) 24 sessions (8 wks)	12.2 ± 0.4 13.6 ± 0.7	10 exercises 75–90%1RM	↔BMI ↑LM ↔BF
Kelly et al. [34]	13 (RT) 13 (C) 32 sessions (16 wks)	15.4 ± 0.9 15.6 ± 0.96	Wks 1–4 1 × 10–15 Light intensity Wks 5–10 2–3 × 13–15 moderate intensity Wks 11–16 3–4 × 8–12 high intensity	↔BF ↔WC ↔LM ↔BMI ↔IS
Benson et al. [39]	33 (RT) 33 (C) 16 sessions (8 wks)	12.3 ± 1.3 12.2 ± 1.3	11 exercises 2 × 8RM	↓WC ↓BF ↓BMI ↑MS
Dorgo et al. [40]	63 (RT) 129 (C) 54 sessions (18 wks)	16.0 ± 1.2 15.8 ± 1.1	2–4 × 8–14	↑MS ↔BMI
Davis et al. [41]	09 (RT) 07 (C) 32 sessions (16 wks)	15.7 ± 1.2 15.1 ± 1.1	62–97%1RM 1–3 sets × 8–15	↔BF ↔LM ↔BMI ↑MS
Velez et al. [42]	13 (RT) 15 (C) 36 sessions (12 wks)	16.1 ± 0.2	07 exercises 2–3 × 10–15RM	↑MS ↑LM ↔BMI ↔BF
Suh et al. [43]	10 (RT) 10 (C) 36 sessions (12 wks)	13.10 ± 0.32 13.10 ± 0.57	10 exercises 2–3 × 10–12RM	↓BF ↔IS ↑WC ↔BMI
Lee et al. [44]	16 (RT) 13 (C) 36 sessions (12 wks)	14.6 ± 1.5 14.8 ± 1.4	10 exercises 2 × 8–12RM	↓BF ↑LM ↓BMI ↑IS ↑CRF
Lee et al. [45]	16 (RT) 12 (C) 36 sessions (12 wks)	14.8 ± 1.9 15.0 ± 2.2	10 exercises 2 × 8–12RM	↑MS ↓ BF ↔BMI
Takai et al. [46]	36 (RT) 58 (C) 45 sessions (8 wks)	13.6 ± 0.6 13.8 ± 0.5	100 reps/day Squat exercise	↔LM ↔BF ↑MS ↔BMI
Sigal et al. [47]	78 (RT) 76 (C) 88 sessions (22 wks)	15.9 ± 1.5 15.6 ± 1.3	07 exercises 3 × 8RM	↓WC ↓BF ↔BMI
Dias et al. [48]	24 (RT) 20 (C) 36 sessions (12 wks)	14.1 ± 1.0 14.7 ± 1.4	12 exercises 1–2 Wks 1 × 10–15 50%–70% 10RM 3–6 Wks 2 × 8–12 60%–80% 10RM 7–12 Wks 3 × 6–10 70%–85% 10RM	↓WC ↓BF ↔LM ↑IS ↔BMI
Horner et al. [49]	27 (RT) 24 (C) 36 sessions (12 wks)	14.6 ± 1.9 14.9 ± 1.8	10 exercises 2 × 8–12RM	↓BF ↑CRF
Yoshimoto et al. [50]	27 (RT) 20 (C) 45 sessions (8 wks)	13.8 ± 0.6 13.8 ± 0.5	100 reps/day Squat exercise	↓BF ↑LM ↔MS ↔BMI
Goldfield et al. [51]	78 (RT) 76 (C) 16 sessions (04 wks)	15.9 ± 1.5 15.6 ± 1.3	07 exercises 3 × 8RM	↑MS
Yetgin et al. [52]	08 (RT) 08 (C) 72 sessions (24 wks)	16.6 ± 1.0 17 ± 0.7	1–8 Wks 50–60%1RM 9–16 Wks 60–70%1RM 17–24 Wks 70–75%1RM	↓ BF ↔BMI ↔LM

The variables presented refer to the authors, population, duration, age, RT and outcomes

*BF* body fat, *BMI* body mass index, *C* control, *CRF* cardiorespiratory fitness, *IS* Insulin sensitivity, *LM* lean mass, *MS* muscle strength, *RM* repetition maximum, *RT* resistance training, *WC* waist circumference, *WKS* weeks, ↑ increase, ↓ decrease, ↔ no change

### Risk of Bias in the Included Articles

**Fig. 3:**
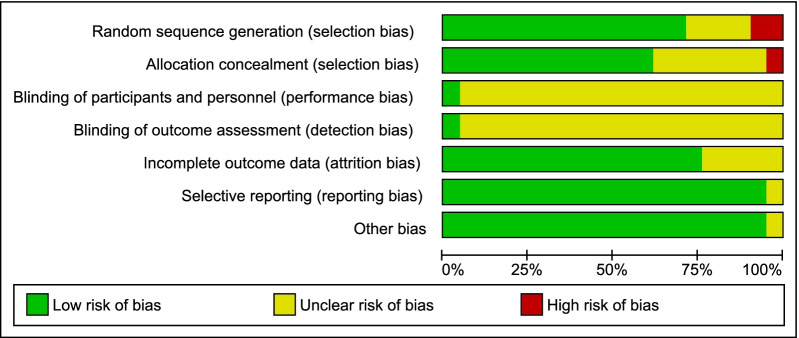
Risk-of-bias item presented as percentages across all included studies

**Fig. 4:**
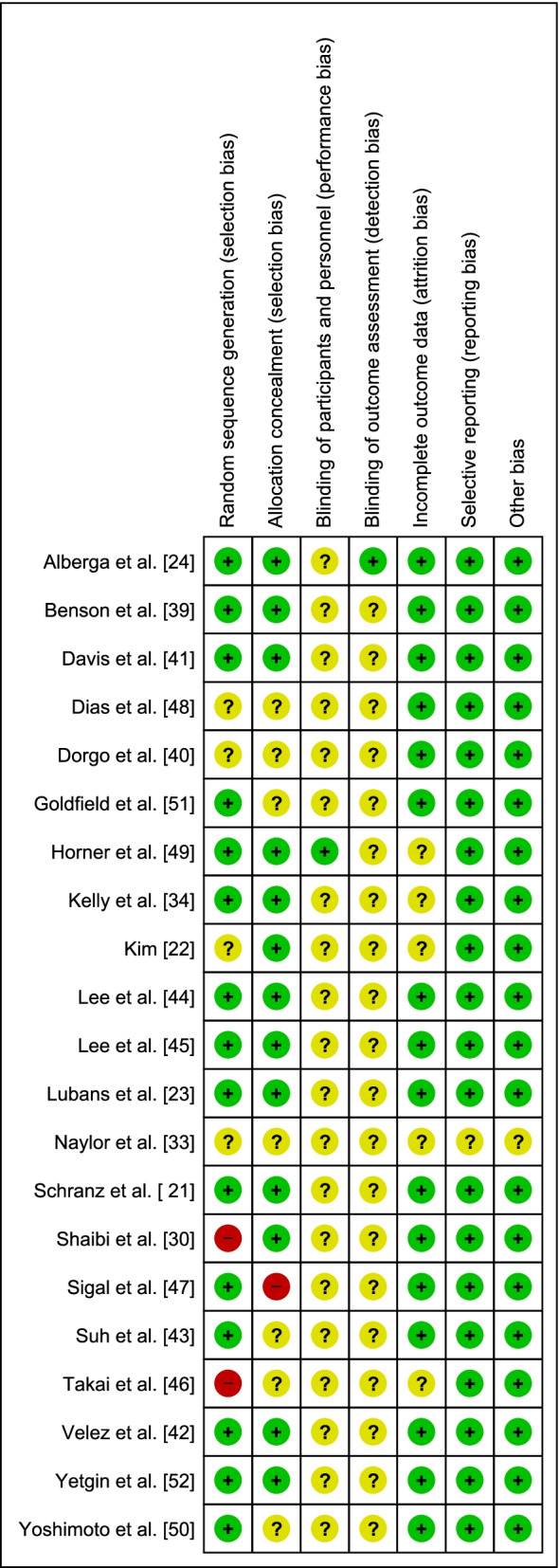
Judgments about each risk-of-bias item for each included study + indicates low risk, ? indicates unclear risk, − indicates high risk

In general, it was possible to notice the low risk and unclear risk of bias in key criteria in most articles. A high percentage of low risk was found in the following key criteria: incomplete outcome data, selective reporting, random sequence, and other biases. A moderate percentage was found in allocation concealment. The studies have revealed a high percentage of unclear risk of bias in blinding participants and personnel and blinding of outcome assessment. In addition, a few studies have revealed a high risk of bias in the generation of random sequence and allocation concealment (Fig. 3 and Fig. 4).

## Discussion

The present review aimed to synthesize the studies that analyzed the effects of RT protocols in obese adolescents, namely on BMI, lean mass, body fat, muscle strength, insulin sensitivity, and cardiorespiratory fitness. Studies that met the inclusion criteria were published mainly between 2006 and 2018, possibly because the interest of health and physical activity professionals in this area of research has been growing rapidly since 2006. The outcomes were consistent with the conclusions achieved in the adult population interventions [53–55] and showed that RT is an effective treatment for obese adolescents. Besides improving muscle strength and cardiorespiratory fitness, RT interventions seem to reduce obesity in adolescents by reducing body fat, waist circumference, and BMI.

Most clinical recommendations for treating obesity are multifactorial, based on a combination of different interventions, such as changing dietary practices, use of medications, and regular physical activity [56, 57]. Several studies reviewed reported favorable variations in body composition after a RT program, namely a significant decrease in body fat [22] and waist circumference [22], an increase in lean mass [42], and a reduction of BMI [22]. Positive effects were also found on muscle strength [21, 23, 24, 30, 39–42, 51] and cardiorespiratory fitness [22, 44, 49] as well as an increase of insulin sensitivity [30, 44]. Despite these positive results in some studies, the standard mean difference did not show a significant effect on lean mass and insulin sensitivity. This could be explained by the small number of studies that analyzed these variables, especially lean mass, resulting in a large heterogeneity statistic (*I*^2^). The different procedures used to evaluate these variables could have also influenced the heterogeneity of results. Moreover, the lack of control of the diet in some protocols and/or the lack of information about feeding may have led to the non-significance of the results, although they tended to be positive.

The analyzed studies used training programs with moderate to submaximal intensities with 1–3 sets of 3–20 repetitions over periods ranging from eight weeks to 1 year. On average, the studies used 2–3 times/week ∼60 min/session for 12 weeks. The data are in line with several recommendations found in the literature [14, 16] indicating that moderate-intensity RT programs should be carried out in the presence of professionals to ensure effective and safe results. The studies included 10–12 exercises per session, using external loads between 70 and 85% of maximal load. They involved mostly large muscle groups (e.g., leg press, bench press, deadlift, lat pull-down, leg extensions, leg curls, squats, push-ups, abdominal crunch, lunges and dumbbell exercises). However, specific exercises were also used but less frequently (e.g., biceps curl, triceps pushdown, calf raise and shoulder press).

Some of the studies that did not reveal a decrease in body fat did not include an eating plan in the intervention protocols, which may be why this variable did not change [21, 33, 34, 42, 46]. Thus, it appears that a RT program aimed at reducing body fat in obese adolescents needs to be combined with a nutritional plan to be more effective [16, 17]. Interestingly, a systematic review published by Hsu et al. [58], in which the authors compared the effect of RT and diet in obese individuals, showed that the diet revealed only positive effects on body fat, with no additional benefits in the performance of physical activities. On the other hand, other findings suggested that RT is associated with increased muscle strength and improved physical performance, essential for reducing obesity and increasing training intensity [59].

No significant reduction in body fat was observed in some studies, yet there was an increase in lean body mass [21, 33, 42]. Despite this, some studies did not reveal changes in lean mass, probably due to the intervention time or using very small samples [34, 48, 52]. Thus, it will be necessary to use protocols with a duration of at least 12 weeks of intervention (2–3 times/week ∼60 min/session) and with 10–12 exercises of intensities of 50–85%1RM for an increase in lean mass to occur in obese adolescents [14, 16]. This variable is important for the control of body weight, directly related to the increase or maintenance of the resting metabolic rate (i.e., lipid oxidation and glucose transport capacity) and with the excess post-oxygen consumption (EPOC) effect resulting in higher daily energy consumption [8, 59, 60].

The results showed a reduction in BMI after RT program. However, this variable was unalterable in some studies, possibly because of the increase in lean mass [30, 42]. In training programs based on resistance exercises, an increase in lean mass is expected, influencing BMI values [61]. Furthermore, the literature shows that RT increases the amount of lean mass in obese young people [17, 58]. The majority of the studies that assessed lean mass also revealed an increase. For instance, Shaibi et al. [30], after a RT program (2 times/week ∼60 min/session), demonstrated an increase in body weight in obese adolescents. However, this increase was caused by gains in lean body mass (54.4 ± 3.2 to 58.1 ± 3.1 kg) as supported by other studies [42, 44]. Nevertheless, evidence is controversial on whether adolescents may increase lean mass after a RT program due to lower testosterone levels when compared to adults [62]. The uncertainty of the effects of RT in lean mass of obese adolescents is clearly evidenced by the results of the current review.

Interestingly, the RT has shown an improvement in insulin sensitivity in some studies [30, 44], supporting the thesis of an effective training strategy for endocrinological regulation [63]. Some authors suggest that these results may be due to isometric contractions that produce insulin-like effects on glucose uptake, with skeletal muscle being the leading site of glucose disposal [43, 64]. Lee et al. [44] stated that after 36 sessions during 12 weeks of RT, improvements in insulin sensitivity, skeletal muscle mass, and muscular strength were achieved. These factors are essential for obese adolescents, as RT is associated with significant reductions in intrahepatic lipids. Fatty liver is a common feature of childhood obesity, and increased hepatic fat is strongly associated with insulin resistance and hypertriglyceridemia in adolescents [65]. Suh et al. [43] also found an improvement in insulin sensitivity after 36 sessions during 12 weeks of RT. However, the RT program was combined with diet, reduced intake of added sugar and increased dietary fiber as in the study of Shaibi et al. [30]. Further investigation is needed to better understand the effect of RT on insulin sensitivity. This variable was only analyzed by five studies in the current review, and no significant change was found when combining all the results.

Regarding cardiorespiratory fitness, the literature indicates that the higher it is, the lower the risk factors for obesity are [66]. Still, AT is often not well tolerated by obese adolescents due to the additional weight they carry [14]. Most studies that analyzed the effects of RT on cardiorespiratory function found an increase in this variable [22, 44, 49], which could be an alternative strategy to AT. Kim [22] revealed an increase of cardiorespiratory fitness (25.8%) after 36 sessions (12 weeks). Lee et al. [44] and Horner et al. [49] also found improvements after a RT program in VO2peak. Ramos-Campo et al. [67] revealed that RT based on circuit training can positively affect cardiorespiratory fitness, namely VO2max, maximum aerobic speed or power, and aerobic performance. Furthermore, two factors have been attributed to RT producing greater EPOC. The first refers to hormonal responses that can change metabolism, specifically catecholamines, cortisol, and growth hormone. The second is tissue damage, which is accompanied by the stimulus for tissue hypertrophy, and after exercise, there is a compensatory phenomenon, and protein synthesis requires high energy demand. This mechanism can also contribute to prolonged stimulation of energy expenditure after exercise [20].

Knowing that waist circumference is related to increased cardiometabolic risk [68], decreasing its dimension during adolescence may carry some prevention because each additional year of abdominal obesity is related to a 4% higher risk of developing diabetes mellitus [69]. Also, Savva et al. [70] described that waist circumference was directly associated with high blood pressure and high lipid concentrations, suggesting that this variable is essential for identifying adolescents with more potential of developing metabolic and cardiovascular diseases. Some authors revealed positive results regarding decreased waist circumference after an intervention of RT in adolescents [22, 34, 39, 47, 48]. For example, Kim [22] found a significant decrease in waist circumference (-2.3 ~ 2.4 cm) during 12 weeks of intervention (3 days/week, 60 min/session, 1–2 × 12/50–60%1RM). Also, Sigal et al. [47], in 22 weeks of RT (4 days/week, 3 × 8RM), showed a significant decrease in this variable (− 2.2 cm). Still, some programs have not found significant results related to this specific parameter [23, 43]. The main reason may have been due to a brief intervention period [23].

Among the analyzed studies, some limitations should be addressed. Several studies used a small number of participants and different intervention durations that could have influenced results. Due to the difference in training programs (e.g., different durations, intensities, and exercises performed), some issues regarding methodological quality (e.g. unclear or high risk of bias in some protocols), and some unclear dietary control of the participants, the results found should be carefully analyzed. Additionally, some studies included boys and girls together in the same group, which may have affected the analysis of the results. It is important to note that the prepubescent and post-pubertal participants included in the review may have impacted the outcomes due to their different maturational statuses. Future investigations should be carefully designed to reduce these limitations and include more variables to better understand the effects of RT interventions in this population (e.g., blood pressure responses, hormonal responses, perceived exertion monitoring during training).

## Conclusions

The RT program seems to be safe and demonstrates benefits in obese adolescents. The literature showed reduced body fat mass, waist circumference and body mass index, and increased muscle strength and cardiorespiratory fitness after a RT program in obese adolescents. Interventions that used protocols for at 2–3 times/week ∼60 min/session during 12 weeks seemed to show more positive effects. The results of our study can help exercise professionals to create training protocols for this type of population. Further investigations are necessary to better understand the role of RT in the body composition of obese adolescents. There is a tendency for participants in RT programs to be enthusiastic and enjoy the exercise intervention, which can be an alternative exercise modality for obese adolescents. In some studies, the increase in lean mass associated with RT influences weight gain. Also, the expected growth of young adolescents can increase weight during the intervention program. Thus, it would be interesting to analyze the minimum training period that affects body composition, excluding the impact of average growth.

## Data Availability

The datasets used and/or analyzed during the current study are available from the corresponding author on reasonable request.

## Abbreviations

AT: Aerobic training
BF: Body fat
BMI: Body mass index
C: Control
CI: Confidence interval
CRF: Cardiorespiratory fitness
EPOC: Excess post-oxygen consumption
IS: Insulin sensitivity
LM: Lean mass
MS: Muscle strength
PRISMA: Preferred reporting items for systematic reviews and meta-analyses
RM: Repetition maximum
RT: Resistance training
SMD: Standardized mean difference
WC: Waist circumference
WKS: Weeks
